# Supplementary material for: Tn‐seq of Thermus thermophilus Genome Reveals Unexpected Tolerance to Insertions in Bacterial Common Essential Genes
Source: Microbiologyopen. 2026 Mar 19;15(2):e70207. doi: 10.1002/mbo3.70207 (PMC13140390; doi:10.1002/mbo3.70207)
Supplement: Supplementary file 1 — Figure S1: TnSeq libraries mapping statistics. Figure S2: Detail coverage of TnSeq libraries on the mayor insertion hotspots, centred in coordinates 576,160 (A) and 1,457,335 (B) of the HB27 chromosome. Figure S3: Clustering methods comparison. Figure S4: Distribution of TnSeq gene groups by standard functional categories. Figure S5: Comparison of TnSeq gene groups and Pangenome analyses. Table S1: List of oligonucleotides. Table S2: Statistics of processed reads of HB27 Tn‐seq libraries from FastQC. See Methods for details. [file MBO3-15-e70207-s003.docx]

**Tn-seq of *Thermus thermophilus* genome reveals unexpected tolerance to insertions in general bacterial essential genes.**

Cristina L. Gómez-Campo, Marc Gost, Bruna Fernanda Silva de Sousa, Laura Álvarez, José Berenguer, Modesto Redrejo-Rodríguez and Mario Mencía

**SUPPLEMENTARY MATERIAL**


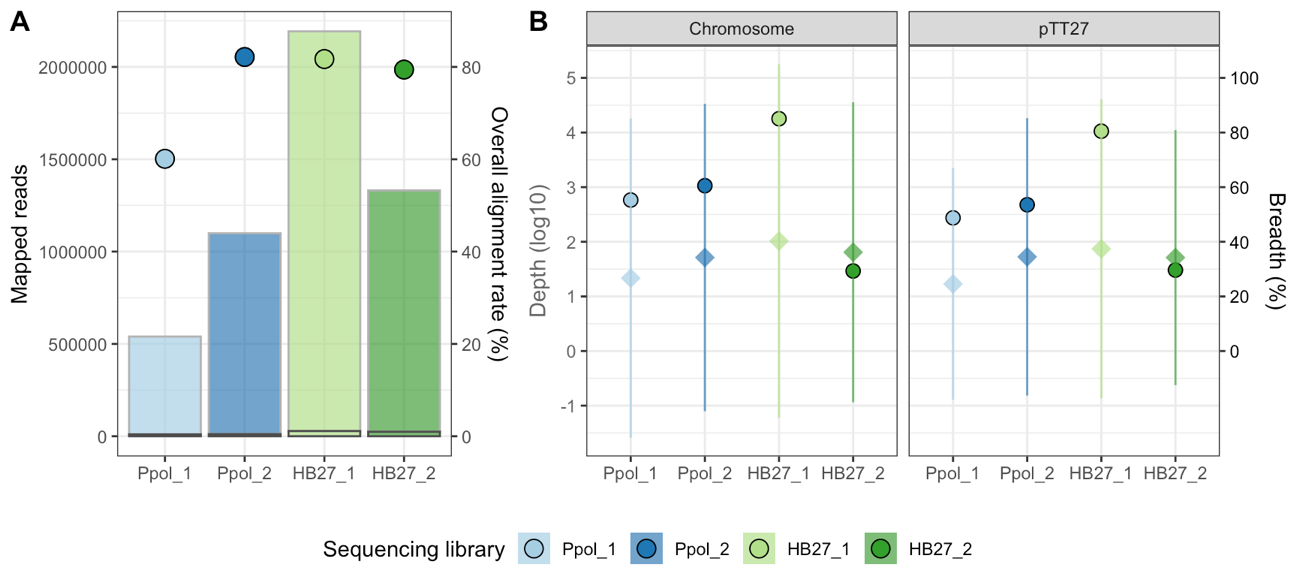


**Figure S1. TnSeq libraries mapping statistics.**

A. Total number of *bowtie2* mapped reads (bars, left axis) and overall alignment rate (points, right axis) in the and Ppol (blue and light blue) HB27 (green and light green) sequenced libraries The ratio of reads that mapped in multiple sites in the reference genome is represented with black squares. B. Tth HB27 chromosome and pTT27 plasmid coverage for each TnSeq library. Plots represent the mean and standard deviation of coverage depth (diamonds, left axis) and genome coverage breadth (points, right axis).

**Figure S2. Detail coverage of TnSeq libraries on the mayor insertion hotspots, centred in coordinates 576,160 (A) and 1,457,335 (B) of the HB27 chromosome.**

For each hotspot, the upper panel shows the coverage depth per nucleotide in the surrounding region for the filtered and trimmed reads of each library. Note the different scale range for each sample. The panels underneath sow the annotated features in the same region. The vertical dashed line indicates the position of the insertion site with the highest coverage.


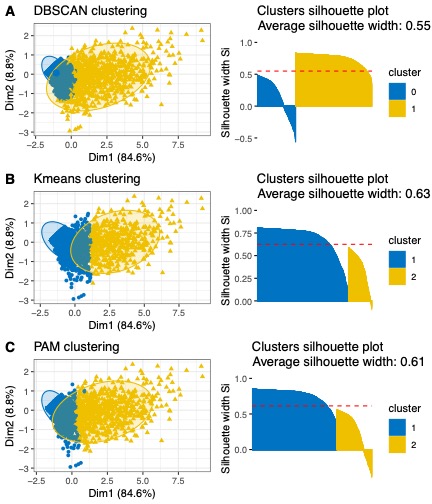


**Figure S3. Clustering methods comparison.**

Cluster results and silhouette plots for DBSCAN (A), K-Means (B) and PAM (C) clustering methods and average silhouette values are shown. The red horizontal dashed line denotes the average silhouette width. The optimal number of clusters in K-means and PAM methods was determined as 2 by the higher average silhouette values, which also coincided with the determination made using the Elbow method.


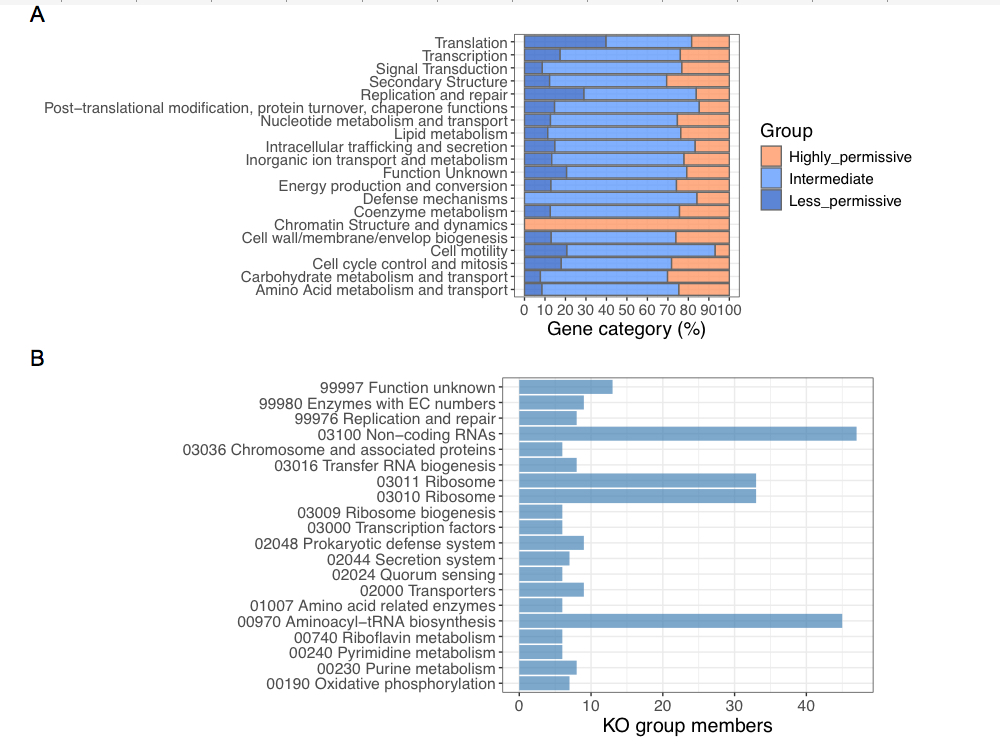


C


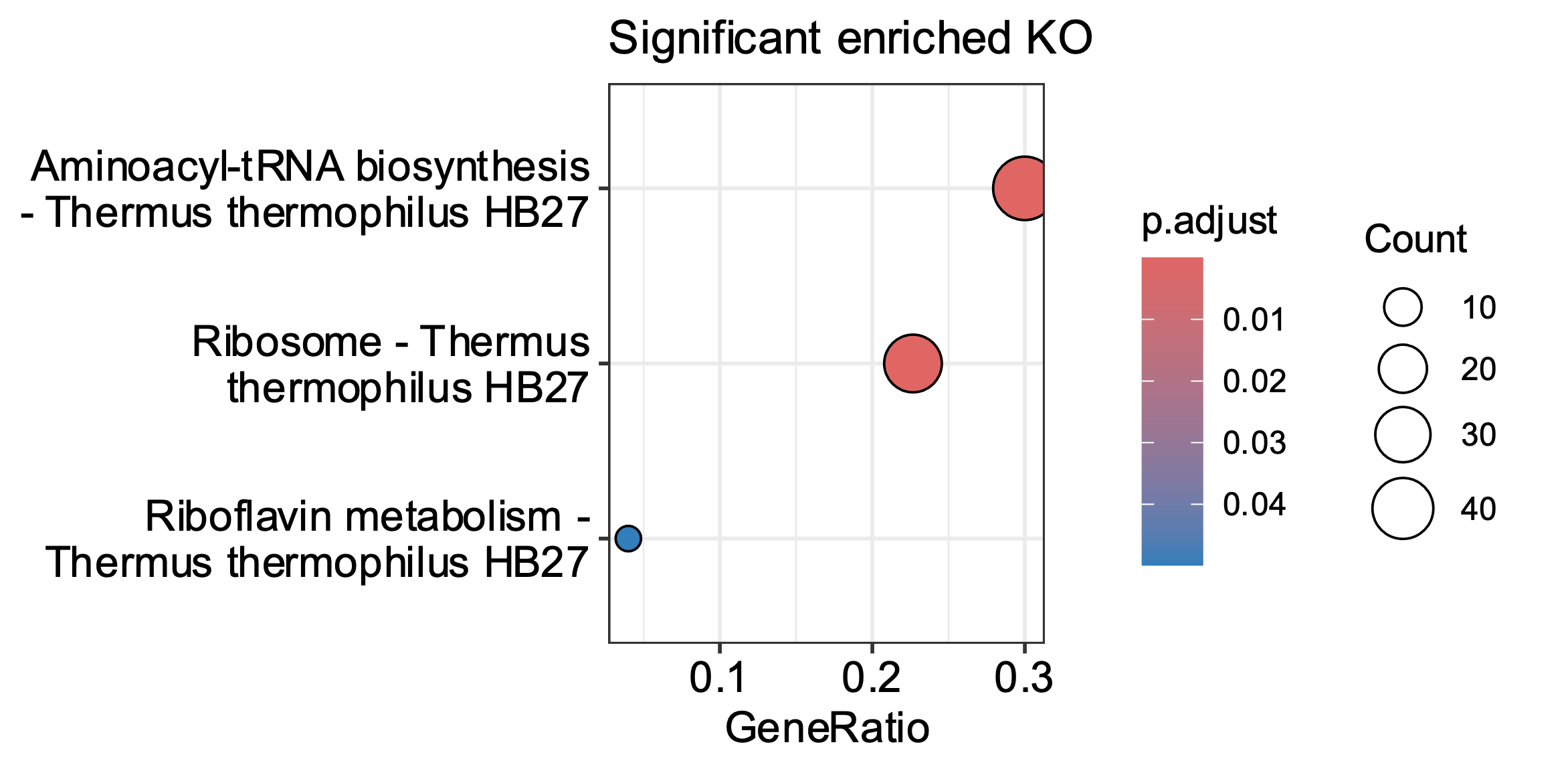


**Figure S4. Distribution of TnSeq gene groups by standard functional categories.**

A. Distribution of annotated main COG categories across TnSeq gene categories. B. More frequent (n>5) KEGG Orthology (KO) genes within the Less Permissive group. Note that COG and KEGG functional annotation could assign functional group to 89.5% and 62.3% of the Tth genes, respectively. C. KO genes enrichment analysis in the Less Permissive shortlisted genes. The bubble size corresponds to the number of all genes within the Less Permissive group with the corresponding term and coloured by adjusted p-value.

**Figure S5. Comparison of TnSeq gene groups and Pangenome analyses.**

Upset plots show the overlapping between HB27 genes classification in each TnSeq and their distribution within pangenome categories for each taxonomic level. The connected dots indicate which groups are being compared. The vertical bars show the number of genes shared between specific combinations of these groups. The horizontal bars indicate the total number of genes in each group.

**Table S1. List of oligonucleotides.** The Mosaic Ends (ME) recognized by Tn5 transposase are in italics. Index Primer R sequence is underlined. NH_2_ denotes that the 3´position of the oligonucleotide has its OH substituted by that group to avoid extension and ligation. P5-InvRep-VarF1-6, is an equimolar mix of primers with the indicated sequence at the NNN variable region, in bold the region complementary to the Mosaic End.

| Oligonucleotides | Sequence (5’-3’) |
| --- | --- |
| *TnKanPslp* | AAA*CTGTCTCTTATACACATCT*AAGCTTGGGATCGATCCCCGGGAGTATAA |
| *TnKanEnd* | AAA*CTGTCTCTTATACACATCT*GAATTCGCGGTATTTCACACCGCATAAATTCC |
| *KatH* | GATCTTCCTTCAGGTTATG |
| *Index Fork Adapter 1* | GTGACTGGAGTTCAGACGTGTGCTCTTCCGATCTGGTCGTGGTAT |
| *Index Fork Adapter 2* | TACCACGACCA-NH_2_ |
| *Index Primer R* | GTGACTGGAGTTCAGACGTGTG |
| *P5-InvRep-VarF1-6* | AATGATACGGCGACCACCGAGATCTACACTCTTTCCCTACACGACGCTCTTCCGATCTNNN**CAGATGTGTATAAGAGACAGT** |
|  | Where NNNN= _ or C or AT or TGTC or TCGAC or GCAGCGAC in an equimolar mix |
| *P7-AD014-index-R* | CAAGCAGAAGACGGCATACGAGATGGAACTGTGACTGGAGTTCAGACGTGTGCTCTTCCGATC |

**Table S2. Statistics of processed reads of HB27 Tn-seq libraries from FastQC. See Methods for details.**

| **Library** | **Reads** | **% Duplication estimation** | **Final Reads (M)** | **Final Reads (%)** | **GC %** |
| --- | --- | --- | --- | --- | --- |
| ***Ppol_1*** | 2170192 | 78.20 | 0.9 | 41.40 | 59.40 |
| ***Ppol_2*** | 1820971 | 78.60 | 1.7 | 92.10 | 64.90 |
| ***HB27_2*** | 3196758 | 72.40 | 2.7 | 83.90 | 65.40 |
| ***HB27_1*** | 1515716 | 74.20 | 1.3 | 88.30 | 65.80 |
